# Supplementary material for: Reconciling Mining with the Conservation of Cave Biodiversity: A Quantitative Baseline to Help Establish Conservation Priorities
Source: PLoS One. 2016 Dec 20;11(12):e0168348. doi: 10.1371/journal.pone.0168348 (PMC5173368; doi:10.1371/journal.pone.0168348)
Supplement: S1 Dataset — (ZIP) [file pone.0168348.s002.zip › Taxa/Serra Norte/SN_2013/N4WS/Lista N4WS-61.pdf]

CAVIDADE N4WS-0061

Classe	Ordem	Fam/Outros	Gên/Outros	Espécie	S-U	U-S	Zon.
Arachnida	Acari	(Parasitengonina)		sp.3	X		E
Arachnida	Acari	(Parasitengonina)		sp.6	X		E
Arachnida	Araneae	Araneidae	*Alpaida*	*septemmammata*		X	E
Arachnida	Araneae	Corinnidae		jovem	1	1	E
Arachnida	Araneae	Oonopidae		jovem		X	E
Arachnida	Araneae	Pholcidae	*Mesabolivar*	*aurantiacus*		X	E
Arachnida	Araneae	Pholcidae	*Mesabolivar*	*eberhard*		X	E
Arachnida	Araneae	Pholcidae		jovem	X		E
Arachnida	Araneae	Prodidomidae	*Lygromma*	sp.2		X	E
Arachnida	Araneae	Salticidae	*Amphidraus*	sp.1		X	E
Arachnida	Araneae	Theridiidae	*Episinus*	sp.1		X	E
Arachnida	Araneae	Theridiosomatidae	*Plato*	sp.1	X	X	E
Arachnida	Araneae	Uloboridae	*Uloborus*	sp.1		X	E
Arachnida	Opiliones	Sclerosomatidae		jovem		X	E
Arachnida	Pseudoscorpiones	Chernetidae		jovem	X		E
Arachnida	Pseudoscorpiones	Chthoniidae	*Pseudochthonius*	sp.1	X		E
Chilopoda	Scolopendromorpha	Scolopocryptopidae	*Dinocryptops*	*miersii*		1	E
Diplopoda	Polydesmida	Pyrgodesmidae		sp.1		X	E
Diplopoda	Polyxenida	Hypogexenidae		sp.1		X	E
Entognatha	Diplura	Campodeidae		sp.1		X	E
Insecta	Blattodea	Blattellidae		jovem	1		E
Insecta	Blattodea	Blattidae		jovem		1	E
Insecta	Blattodea	Polyphagidae		jovem		1	E
Insecta	Diptera	Chironomidae		sp.	X		E
Insecta	Diptera	Culicidae	*Culex*	sp.		X	E
Insecta	Diptera	Ephydridae		sp.		X	E
Insecta	Diptera	Psychodidae	*Evandromyia*	*saulensis*		X	E
Insecta	Diptera	Psychodidae	*Sciopemyia*	*sordellii*	X		E
Insecta	Diptera	Tipulidae	Tipulinae	sp.	X		E
Insecta	Heteroptera	Reduviidae	Reduviinae	jovem	7	4	E
Insecta	Hymenoptera	Formicidae	*Apterostigma*	sp.1		X	E
Insecta	Hymenoptera	Formicidae	*Nylanderia*	sp.3		X	E
Insecta	Hymenoptera	Formicidae	*Pachycondyla*	*striata*		1	E
Insecta	Hymenoptera	Formicidae	*Solenopsis*	sp.2		X	E
Insecta	Isoptera	Termitidae	*Cortaritermes*	*silvestrii*	X		E
Insecta	Isoptera	Termitidae	*Nasutitermes*	sp.		X	E
Insecta	Lepidoptera	Gelechioidea		sp.4		X	E
Insecta	Lepidoptera	Noctuoidea		sp.6		X	E
Insecta	Lepidoptera	Tineoidea		sp.6		X	E
Insecta	Neuroptera	Myrmeleontidae		jovem	21	2	E
Insecta	Orthoptera	Phalangopsidae	*Paraclodes*	sp.		7	E
Insecta	Orthoptera	Phalangopsidae	*Phalangopsis*	sp.	35		E
Insecta	Psocoptera	Psyllipsocidae		jovem		X	E
Amphibia	Anura			sp.3	1		E
Mammalia	Chiroptera	Emballonuridae	*Peropteryx*	sp.		3	E
Mammalia	Rodentia	Cricetidae	*Rhipidomys*	sp.	1		E
Reptilia	Squamata	Gekkonidae	*Thecadactylus*	sp.		1	E
